# Economic cost and quality of life of family caregivers of schizophrenic patients attending psychiatric hospitals in Ghana

**DOI:** 10.1186/s12913-017-2642-0

**Published:** 2017-12-04

**Authors:** Yaw Nyarko Opoku-Boateng, Irene A. Kretchy, Genevieve Cecilia Aryeetey, Duah Dwomoh, Sybil Decker, Samuel Agyei Agyemang, Yesim Tozan, Moses Aikins, Justice Nonvignon

**Affiliations:** 1 National Health Insurance Authority, Accra, Ghana; 2 Department of Pharmacy Practice and Clinical Pharmacy, School of Pharmacy, College of Health Sciences, University of Ghana, Legon, Ghana; 3 Department of Health Policy, Planning and Management, School of Public Health, College of Health Sciences, University of Ghana, Legon, Ghana; 4 Department of Biostatistics, School of Public Health, College of Health Sciences, University of Ghana, Legon, Ghana; 5 Pantang Psychiatric Hospital, Accra, Ghana; 6 College of Global Public Health, New York University, New York, NY USA

**Keywords:** Economic burden, Quality of life, Caregiving, Schizophrenia, Ghana

## Abstract

**Background:**

Low and middle income countries face many challenges in meeting their populations’ mental health care needs. Though family caregiving is crucial to the management of severe mental health disabilities, such as schizophrenia, the economic costs borne by family caregivers often go unnoticed. In this study, we estimated the household economic costs of schizophrenia and quality of life of family caregivers in Ghana.

**Methods:**

We used a cost of illness analysis approach. Quality of life (QoL) was assessed using the abridged WHO Quality of Life (WHOQOL-BREF) tool. Cross-sectional data were collected from 442 caregivers of patients diagnosed with schizophrenia at least six months prior to the study and who received consultation in any of the three psychiatric hospitals in Ghana. Economic costs were categorized as direct costs (including medical and non-medical costs of seeking care), indirect costs (productivity losses to caregivers) and intangible costs (non-monetary costs such as stigma and pain). Direct costs included costs of medical supplies, consultations, and travel. Indirect costs were estimated as value of productive time lost (in hours) to primary caregivers. Intangible costs were assessed using the Zarit Burden Interview (ZBI). We employed multiple regression models to assess the covariates of costs, caregiver burden, and QoL.

**Results:**

Total monthly cost to caregivers was US$ 273.28, on average. Key drivers of direct costs were medications (50%) and transportation (27%). Direct costs per caregiver represented 31% of the reported monthly earnings. Mean caregiver burden (measured by the ZBI) was 16.95 on a scale of 0–48, with 49% of caregivers reporting high burden. Mean QoL of caregivers was 28.2 (range: 19.6–34.8) out of 100. Better educated caregivers reported lower indirect costs and better QoL. Caregivers with higher severity of depression, anxiety and stress reported higher caregiver burden and lower QoL. Males reported better QoL.

**Conclusions:**

These findings highlight the high household burden of caregiving for people living with schizophrenia in low income settings. Results underscore the need for policies and programs to support caregivers.

## Background

Mental illness accounts for about 32% of years lived with disability (YLD) and about 13% of disability-adjusted life-years (DALYs), a percentage that equals that of cardiovascular and circulatory diseases [1]. Schizophrenia is a severe mental disorder characterized by significant distortions in thinking and perception, accompanied by an exhibition of inappropriate emotions [2, 3]. Schizophrenia alters individuals’ perception of reality, often making them think and act in ways that are strange or abnormal by socially sanctioned standards. The World Health Organization (WHO) estimates that about 21 million people suffer from schizophrenia globally [4]. It is estimated that 13% of Ghanaians suffer from at least one form of mental or substance abuse disorder and that about 32% of all mental health cases managed in the country’s three psychiatric hospitals are schizophrenic [5].

Caregiving plays a significant role in the management of schizophrenia. For example, the WHO indicates that globally up to 90% of schizophrenic patients live with their families [6]. In many low and middle income countries (LMICs), the social structure of families and economic hardship impede the use of paid caregivers. Other health systems challenges, such as inadequate health personnel and poor infrastructure, further increase the burden of caring for people with mental disorders.

A key provision of Ghana’s 2012 Mental Health Act (Act 846) is to de-institutionalize mental health care (i.e., provide community-based care for people with mental disorders) in order to decongest the three psychiatric hospitals. The role of caregivers in this community-based approach is crucial since family interactions influence treatment outcomes and relapse rates [7]. However, as in other LMICs, family caregivers of schizophrenic patients in Ghana bear significant economic, psychological and social burdens, which are often unaccounted for in interventions [8–15]. For instance, the time required to care for schizophrenic patients affects the productivity of caregivers, often limiting their participation in the labor force, which has economic implications for the family. Families also incur substantial medical and non-medical costs associated with the management of the condition, which may lead to financial impoverishment. In addition, caring people with mental disorders places significant psychological burden on caregivers and has been shown to negatively affect caregivers’ quality of life (QoL) [16, 17]. Other social burdens that families bear include stigma, which affects the social support that is much needed for such patients and their families.

Although Ghana has a high burden of mental illness, few studies have examined the burden of mental illness in general—and schizophrenia in particular—on unpaid family caregivers. In this study, we sought to address this gap by estimating the economic burden and quality of life of family caregivers of schizophrenic patients in Ghana.

## Methods

### Study setting

The study was undertaken at the three psychiatric hospitals in Ghana: Accra, Pantang and Ankaful. Accra Psychiatric Hospital is a 600-bed hospital commissioned in 1906 as the first psychiatric hospital in Ghana and is located in the Greater Accra Region. Established in 1975 with a bed capacity of 500, the Pantang Psychiatric Hospital is situated in Pantang, also in the Greater Accra Region. Ankaful Psychiatric Hospital, which was built in 1965, is a 350-bed hospital located near the coastal town of Cape Coast in the Central Region of Ghana that serves patients from the Central, Western and Ashanti regions of Ghana and some neighbouring countries. All the hospitals are located in the southern part of the country and serve the country’s population of approximately 25 million [18].

### Study population and sample size

The study population comprised family (primary) caregivers of schizophrenic patients reporting to the outpatient department at each study site. Based on Cochran [19], the sample size was calculated using the formula below:

$$ {n}_0=(Deff)\frac{{\left({Z}_{\frac{\propto }{2}}\right)}^2p\left(1-p\right)}{e^2} $$

Where *n*_0_ is the minimum required sample size; *Z* ^2^ is an abscissa of the curve that cuts off an area ∝ at the tail (1 – α equals the desired confidence level, i.e., 95%); *e* is the desired level of precision; *p* is the estimated proportion of schizophrenic patients that is present in the population, which was assumed to be 50% since the current proportion is unknown; and *Deff* is the design effect. For a 95% confidence interval, ${Z}_{\frac{\propto }{2}}$ is 1.96 and the level of precision *e* (margin error for the study)±5%. Assuming a design effect of 1.03, we computed a minimum sample size of.

$$ {n}_0={1.03}^{\ast}\frac{1.96^2\times 0.5\times 0.5}{0.05^2}=395.684\approx 396. $$

Assuming a 90% response rate, we estimated a minimum sample size of 440 patients. We computed the sample size required in each facility using probability proportionate to size (PPS) of number of schizophrenic patients (Table 1).

**Table 1:** Sample size for study sites

Hospital	Number of patients	Sample size	Percentage
Accra	11,256	228	51.8
Pantang	6780	138	31.4
Ankaful	3660	74	16.8
Total	21,696	440	100.0

In each hospital, folders of patients diagnosed with schizophrenia at least six months preceding the study were sorted and reviewed using criteria outlined in the 10^th^ revision of the International Statistical Classification of Diseases and Related Health Problems (ICD-10), and primary caregivers who attended the hospital with the patients identified and interviewed. Accompanying relatives who were not the primary caregivers were excluded. Given that many patients presenting for outpatient services were unaccompanied, accompanying primary caregivers were recruited until the required sample size for the particular facility was met.

### Data collection and tools

Data were collected using a structured, interviewer-administered questionnaire with closed- and open-ended questions. The questionnaire elicited information on caregivers’ sociodemographic characteristics, direct health care costs (medical and non-medical costs) and indirect costs (i.e. productivity losses) to caregivers.

### Variables

We used a cost of illness analysis approach. Costs were analysed from the caregiver perspective and included costs incurred during the month preceding the data collection. All costs were computed in United States Dollars (US$) (3.9 Ghanaian Cedi (GHS) ≈ 1 US$). Costs were not adjusted for inflation. Costs were categorized into direct, indirect and intangible costs. Direct costs were further grouped into medical and non-medical costs. Medical costs included the costs of drugs, consultations, laboratory investigations and diagnostics and other therapies. Non-medical costs included the cost of travel to and from hospital, food costs incurred by the caregiver during the treatment period, accommodation costs if the caregiver travelled with the patient away from home, and miscellaneous costs such as telephone costs related to medical care. The sum of medical and non-medical costs constituted the direct costs.

*Indirect costs* were estimated using the national daily minimum wage of US$ 2.0 per day (May 2016) for caregivers in the formal sector and a local daily agricultural wage rate of US$ 4.50 was used for caregivers working in the informal sector. Unemployed caregivers were considered part of the informal sector. Travel time was calculated by adding the total number of hours spent travelling to and from the hospital. Waiting time constituted the total time spent waiting for and receiving treatment (i.e., from the time the caregiver and patient arrived at the hospital to the time they left the hospital). The time used for other caregiving activities, such as household activities and leisure, was also calculated and included in the indirect costs. Total indirect cost was calculated as the product of the sum of total time spent on personal care for patient, transport and waiting for health care, other household activities for the patient (in hours) and the respective (hourly) wage rate. Total economic cost constituted the sum of direct and indirect costs.

Intangible costs (sometimes referred to as caregiver burden) were assessed using the 12-item Zarit Burden Interview (ZBI) tool developed by Bedard et al. [20], which comprises items assessing stress, pain, anxiety and depression. The intangible cost was analysed by summing the scores for all 12 items for each respondent. The overall score ranges between 0 and 48. Scores were then categorized as low burden (0–16) or high burden (17–48).

Quality of life was assessed using the abridged WHO Quality of Life (WHOQOL-BREF) tool. The WHOQOL-BREF comprises 26 items: two items assessing the overall quality of life and general health; and 24 assessing satisfaction in four main domains – seven items on physical health, six items on psychological health, three items on social relationships, and eight items on environmental health. Each item was rated on a scale of 1 to 5 with 1 being the lowest and 5 being the highest QoL. The mean score of items within each domain was used to calculate the domain mean. The mean score for each domain was then multiplied by 4 (four) to make it comparable with the full WHOQOL tool (WHOQOL-100) [21]. The scores were scaled in a positive direction so that a higher score indicated a higher QoL.

Other variables used in the analysis included *Age* (in years) as at last birthday; *Sex* measured as a dummy, 1 for male and 0 for female; *Marital status:* Married (1), single (0); *Proximity of residence from health facility visited;* 1 for far (living more than 30 min from facility, by typical mode of transport used) and 0 for close; Caregiver’s *highest level of education:* no education (0), primary/junior high school (i.e. basic education or equivalent) (1), secondary (2) and tertiary (3); Caregiver’s *employment status:* unemployed (0), self-employed (1), private sector (2), public sector (3), student/apprentice (4); Caregivers were asked whether other members of the family spent time taking care of the patient *(other family support):* yes (1), no (0); and Caregiver’s *mental health status* (i.e. depression, anxiety, stress). Mental health status was assessed using the Depression Anxiety Stress Scale (DASS-21), which comprises of three subscales: Depression (DASS-D), Anxiety (DASS-A), and Stress (DASS-S). Responses on each item ranged from 0 (did not apply to me at all) to 3 (applied to me very much).

Reliability of the QoL and Zarit burden scale was assessed using Cronbach’s alpha. With the exception of the depression sub-scale of the DASS-21 and the social sub-scale of the WHOQOL, the Cronbach alpha values fell within the acceptable range of alpha values (0.70 to 0.95) [22–24] (Table 2).

**Table 2:** Internal consistency of the mental health status, quality of life, and Zarit burden indices

Domain	Number of items	Cronbach’s alpha
Mental health status		
Depression	7	0.67
Anxiety	7	0.80
Stress	7	0.83
Quality of life		
Physical	7	0.82
Psychological	6	0.77
Social	3	0.56
Environmental	8	0.81
Zarit burden index	12	0.83

### Data analysis

Descriptive statistics (mean, standard deviation) of study variables are presented in Table 3. Two sets of multivariate linear regression models were fitted to assess factors associated with economic costs and QoL. The first set of models had direct and indirect costs as dependent variables, and demographic and socioeconomic factors (e.g., age, sex, marital status, proximity of residence to facility, highest education level and employment status) as explanatory variables. The second set of models had the four domains of QoL as dependent variables. Explanatory variables comprised direct costs, indirect costs, Zarit burden scores, time and duration of care provided, mental health indices (anxiety, depression, stress). The models also controlled for caregivers’ sociodemographic characteristics. A final model was fitted to investigate the covariates of caregiver burden (measured by the ZBI score).

**Table 3:** Socio-demographic characteristics of caregivers (*N* = 444)

Characteristic	Number	(%)
Sex		
Male	193	43.5
Female	251	56.5
Age		
< 20	3	0.7
20–29	51	11.5
30–39	95	21.4
40–49	103	23.2
50–59	89	20.0
60–69	76	17.1
> 69	27	6.1
Marital Status		
Married	294	66.2
Single	150	33.8
Religion		
Christian	393	88.5
Muslim	45	10.1
Traditionalist	2	0.5
Other	4	0.9
Educational Level		
No education	50	11.3
Primary	146	32.8
Secondary	149	33.6
Tertiary-Graduate/Post Graduate	99	22.3
Employment Status		
Self employed	240	54.2
Private sector	70	15.8
Public sector	41	9.2
Unemployed	78	17.6
Student/Apprentice	14	3.2

## Results

### Background characteristics of caregivers

Caregivers’ sociodemographic characteristics are summarized in Table 3. Caregivers’ mean age was 47 years (45% were aged 30–49 years and 37% aged 50–69 years). Fifty-seven percent of caregivers were female and 66% were married. Twenty-two percent of the caregivers had tertiary education: 11% had university degrees, and 11% had certificate, diploma or post-diploma qualifications. About 34% of the caregivers had secondary education and 11% had no formal education. In terms of employment status, 54% of caregivers were self-employed, 16% were working in the private sector, 9% were employed in the public sector, and 18% were unemployed. About 3% of the caregivers were students or apprentices.

### Economic costs of caregiving

The average caregiving cost per month was US$273.28, with indirect costs being $242.95 and direct costs being $30.36 (Table 4). Total cost for the study sample was $76,839.54, with indirect costs accounting for about 82.5%. The key components of direct costs were drug (about 50%) and transportation (27%) costs.

**Table 4:** Economic costs to caregiving for schizophrenia

Cost component	N	Total cost^a^ (US$)	Standard deviation	Cost profile^b^ (%)	Average cost (US$)
Direct costs					
Direct medical cost					
Consultation	444	1411.02	7.02	1.8	3.17
Drugs	444	6668.49	22.59	8.7	15.01
Lab/Diagnostics	442	428.20	5.38	0.5	0.97
Other	442	144.64	4.20	0.2	0.33
Sub total		8652.36		11.2	19.50
Direct non- medical costs					
Transportation	444	3675.57	9.62	4.8	8.28
Meals	442	843.15	5.56	1.1	1.90
Lodging	442	153.20	5.05	0.2	0.35
Miscellaneous	442	142.69	1.20	0.2	0.33
Sub total		4814.62		6.3	10.85
Total direct costs		13,466.97		17.5	30.36
Indirect costs					
Formal sector	131	9118.97	40.26	11.9	69.61
Informal sector	313	54,253.59	88.38	70.6	173.33
Total indirect costs	444	63,372.56		82.5	242.95
Grand Total Cost		76,839.54		100	273.28

^a^US$ 1.00 equivalent to GHS 3.9 (Bank of Ghana average monthly interbank exchange rate, June 2016)

^b^Percentages computed by dividing the total cost for the item by the grand total cost and multiplying the result by 100

### Intangible costs of caregiving

The mean caregiver burden score was 16.95 (SD 8.82) out of a maximum of 48. About 51% of the caregivers reported low burden (i.e., ZBI score of >16). The analysis also revealed that the burden in caregiving was significantly higher for females (61%) than for males (39%) among caregivers reporting high burden.

### Caregivers’ quality of life

The mean quality of life scores by domain and background characteristics are summarized in Table 5. The average overall QoL was 28.2 (SD 12.0). Average scores in the four domains were 19.6 (physical), 29.1 (psychological), 29.2 (social), and 34.8 (environmental). QoL scores across all domains were, on average, higher among males (29.6) than females (27.1) (*p* < 0.05). Caregivers who were married had higher QoL scores than those who were unmarried. The mean score across all domains increased as the caregiver’s educational level increased.

**Table 5:** Mean quality of life scores, by domain and background characteristics

	Physical		Psychological		Social		Environmental		Total	
	Mean (SD)	*p*-value	Mean (SD)	*p*-value	Mean (SD)	*p*-value	Mean (SD)	*p*-value	Mean (SD)	*p*-value
Age^a^		0.303		0.257		<0.05		0.434		0.511
< 30	20.1 (17.5)		31.6(24.1)		35.2(27.0)		33.6(23.3)		30.1(13.3)	
30–39	20.4 (17.6)		32.2(25.7)		30.2(24.6)		31.1(24.3)		28.5(11.7)	
40–49	19.7 (15.9)		26.2(22.2)		27.6(24.7)		37.2(25.9)		27.8(11.3)	
50–59	18.8 (14.4)		25.9(21.1)		23.4(21.2)		38.1(25.1)		26.6(12.1)	
60+	19.3 (15.5)		30.6(24.9)		31.6(24.6)		33.6(24.3)		28.8(12.2)	
Sex ^b^		<0.001		<0.01		<0.05		<0.05		<0.05
Male	20.0 (16.1)		30.8(25.0)		31.0(26.3)		36.4(26.1)		29.6(12.6)	
Female	19.3 (16.1)		27.9(22.7)		27.7(22.9)		33.6(23.8)		27.1(11.5)	
Marital status^b^										
Married	20.0(15.7)		30.0(24.1)		29.1(23.6)		33.6(24.7)		28.1(11.7)	
Single	19.0(16.4)	0.270	28.1(23.1)	0.330	29.4(26.1)	0.071	37.2(24.9)	0.436	28.4(12.5)	0.863
Religion^c^		<0.05		0.872		0.486		0.270		0.972
Christian	19.1(15.4)		28.8(23.4)		29.6(24.6)		35.1(24.9)		28.2(11.9)	
Muslim	23.9(20.3)		31.6(26.6)		25.8(23.4)		32.5(24.4)		28.513.0)	
Education^a^		<0.01		<0.001		<0.001		<0.001		<0.001
None	19.1(16.4)		22.3(17.5)		23.2(19.4)		27.7(21.3)		23.1(8.4)	
Primary	19.5(17.9)		26.2(20.2)		23.5(22.1)		30.6(24.7)		24.9(11.5)	
Secondary	20.0(16.2)		28.3(22.5)		30.3(24.7)		35.4(23.7)		28.5(12.0)	
Tertiary	19.1(14.8)		36.1(29.1)		31.9(26.5)		38.9(28.3)		31.5(12.7)	
Employment^a^		0.703		0.140		0.911		0.247		0.558
Unemployed	21.4(19.0)		31.6(25.6)		32.0(25.2)		32.7(23.8)		29.4(13.8)	
Self-employed	19.6(15.7)		27.3(22.2)		29.0(24.6)		35.2(25.3)		27.8(11.3)	
Private sector	17.4(12.5)		32.2(26.4)		27.7(24.4)		38.0(26.1)		28.8(11.8)	
Public sector	19.6(16.3)		28.3(23.1)		26.6(22.5)		31.5(21.7)		26.6(12.3)	
Total	19.6 (16.0)		29.1(23.7)		29.2(24.5)		34.8(24.8)		28.2(12.0)	

^a^Kruskal-Wallis test used in determining significance of difference

^b^Wilcoxon Rank Sum test used in determining the significance of difference

^c^Other religions constituted 1% and was not considered in the analysis due to its insignificance

### Predictors of costs and QoL

The multivariate linear multiple regression results for covariates of economic cost are shown in Table 6. Tertiary education was significantly associated with indirect costs. Compared to those with no formal education, indirect cost for caregivers with tertiary education were lower by GHS 216 (US$55.38, − 95% CI: -329.41, −102.10, *p*-value = 0.0007). A unit increase in age, resulted in a GHS 3.3 increase in indirect cost (US$ 0.75, 95% CI: 0.99, 5.52, *p* < 0.05). Indirect cost for males was GHS 61.1 lower compared to females (US$11.86, 95% CI: -121.77, −0.41, *p* < 0.05).

**Table 6:** Multivariate linear multiple regression results for covariates of economic cost

	Cost			
	Direct	Indirect	Joint effect	
Covariates	*β* (95% CI)	*β* (95% CI)	F-test statistic	*p*-value
		**		
	0.71	3.25		
Age in years	(−0.17,1.59)	(0.99,5.52)	10.18	0.0001***
Sex		*		
Female	*Ref*	*Ref*		
Male	12.06	−61.09	3.65	0.0269*
	(−11.58,35.70)	(−121.77,-0.41)		
Marital status				
Single	*Ref*	*Ref*		
Married	19.30	53.42	3.93	0.0203*
	(−5.58,44.17)	(−10.43,117.28)		
Proximity	*			
Close	*Ref*	*Ref*		
Far	32.33	0.84	2.91	0.0554
	(5.52,59.15)	(−68.00,69.67)		
Education		***		
None	*Ref*	*Ref*		
Primary/junior high school	12.27(−26.56,51.11)	−64.17(−163.87,35.52)	18.61	0.0001***
Secondary	16.19(−22.77,55.16)	−79.76(−179.78,20.27)		
Tertiary	39.84(−4.43,84.11)	−215.75(−329.41,-102.10)		
Employment status		***		
None	*Ref*	*Ref*		
Private	1.07(−40.83,42.96)	−327.68(−435.22,-220.14)		
Public	5.58(−41.69,52.86)	−302.55(−423.91,-181.19)	2.36	0.0953
Self-employed	7.35(−25.48,40.17)	−45.51(−129.79,38.76)		
Student/apprentice	66.56(−6.26,139.38)	−19.64(−206.58,167.30)		
*R* ^2^	50.8%	64.2%		

*β* is the estimated effect of covariate; *R* ^2^ is the adjusted coefficient off determination; CI is confidence interval; *ref.* is the reference category

*p < 0.05, ** *p* < 0.01, *** *p* < 0.001

With respect to predictors of ZBI score, anxiety, depression and stress were related to Zarit burden score, as presented in Table 7. The burden of caregivers with severe anxiety was approximately 4% (95% CI: 1.02, 6.59; *p* = 0.0022) higher than those with no anxiety disorders, controlling for other covariates.

**Table 7:** Multiple linear regression results of total caregiver burden (ZBI)

Covariates	*β*	95% CI
Age in years	−0.01	(−0.07, 0.05)
Sex		
Female	*Ref*	
Male	−0.42	(−1.89, 1.05)
Marital status:		
Single	*Ref*	
Married	−0.09	(−1.64, 1.46)
Proximity		
Close	*Ref*	
Far	0.90	(−0.78, 2.58)
Education		
None	*Ref*	
Primary/junior high school	0.10	(−2.34, 2.55)
Secondary	−4.43	(−2.92, 2.06)
Tertiary	−1.01	(−3.86, 1.84)
Employment status**		
None	*Ref*	
Private	3.47	(0.74, 6.19)
Public	6.09	(3.10, 9.07)
Self-employed	1.79	(−0.30, 3.88)
Student/apprentice	0.17	(−4.37, 4.71)
Direct cost	0.01	(−0.001, 0.01)
Indirect cost	0.002	(−0.002, 0.01)
Duration of care given	0.002	(−0.01, 0.01)
Daily care given time in hours	−0.10	(−0.32, 0.13)
Other family		
No	*Ref*	
Yes	0.42	(−1.03, 1.87)
Anxiety**		
Normal	*Ref*	
Moderate	2.82	(1.05, 4.59)
Severe	3.80	(1.02, 6.59)
Depression***		
Normal	*Ref*	
Moderate	3.24	(1.54, 4.94)
Severe	8.25	(4.71, 11.80)
Stress***		
Normal	*Ref*	
Moderate	5.08	(2.91, 7.24)
Severe	7.70	(4.70, 10.70)
*R* ^2^	51.9	

*β* is the estimated effect of covariate; *R* ^2^ is the adjusted coefficient off determination; *CI* is confidence interval; *ref.* is the reference category

*p < 0.05, **p < 0.01, ***p < 0.001

Table 8 shows that for a unit increase in the Zarit burden score, QoL in relation to the physical domain decreased by 0.25 (95% CI: -0.38, −0.13, *p* = 0.0001). Similar results were obtained for the psychological QoL domains. The coefficients for the Zarit burden score, taken for all the four QoL domains together, were statistically significant (*F* = 5.30, *p* = 0.0004). There was no statistically significant relationship between the direct and indirect cost and the four QoL domains. The QoL in relation to the physical and psychological domains was higher among caregivers who reported family support. The QoL with reference to the psychological domain was about 3% (95% CI: 0.68, 5.12; *p* = 0.0060) higher for participants who reported family support compared to those that did not. Depression was associated with all the four domains of QoL (*F* = 19.20, *p* = 0.0001). The QoL was 17% (95% CI: -22.92, −11.87; *p*-value = 0.0001) lower for participants with severe forms of depression compared to those with no depression. The results further showed that more highly educated caregivers reported higher QoL (7–15% more) than those who were uneducated, and male caregivers reported higher QoL (2–6% more) than female caregivers.

**Table 8:** Multivariate multiple regression results for covariates of quality of life domains

	Domains measuring quality of life					
	Physical	Psychological	Social	Environment	Joint effect	
Covariates	*β* (95% CI) p-value	*β* (95% CI) p-value	*β* (95% CI) p-value	*β* (95% CI) *p*-value	F-test statistic	*p*-value
	−0.14	−0.09	−0.06	−0.05		
Age in years	(−0.22, −0.06)	(−0.18, 0.002)	(−0.17, 0.04)	(−0.13, 0.04)	3.19	0.0133*
Sex	***	**	***	*		
Female	*Ref*	*Ref*	*Ref*	*Ref*		
Male	4.08(2.15, 6.01)	2.93(0.68, 5.17)	5.66(3.09, 8.22)	2.22(0.16, 4.28)	6.14	0.0001***
Marital status:			***			
Single	*Ref*	*Ref*	*Ref*	*Ref*		
Married	0.45(−1.59, 2.48)	−1.73(−4.10, 0.63)	−5.68(−8.38, −2.98)	−2.18(−4.35, −0.02)	5.08	0.0005***
Proximity	***	*	*	**		
Close	*Ref*	*Ref*	*Ref*	*Ref*		
Far	−5.88(−8.10, −3.66)	−3.01(−5.59, −0.43)	−3.57(−6.52, −0.63)	−3.66(−6.02, −1.30)	6.68	0.0001***
Education	***	***	*	***		
None	*Ref*	*Ref*	*Ref*	*Ref*		
Primary/junior high school	2.20(−1.00, 5.40)	3.59(−0.13, 7.31)	6.79(2.54, 11.05)	5.24(1.83, 8.65)		
Secondary	3.02(−0.24, 6.29)	5.12(1.33, 8.91)	5.23(0.90, 9.57)	6.53(3.06, 10.01)		
Tertiary	7.05(3.32, 10.79)	11.04(6.69, 15.38)	9.81(4.84, 14.77)	14.55(10.57, 18.53)	12.03	0.0001***
Employment status						
None	*Ref*	*Ref*	*Ref*	*Ref*		
Private	2.41(−1.18, 6.01)	0.07(−4.11, 4.25)	−1.61(−6.39, 3.16)	1.49(−2.35, 5.32)		
Public	1.12(−2.86, 5.11)	−1.79(−6.42, 2.84)	−5.02(−10.32, 0.27)	−1.26(−5.50,2.99)		
Self-employed	2.81(0.06, 5.56)	1.57(−1.63, 4.76)	−0.90(−4.55, 2.75)	2.86(−0.07, 5.79)		
Student/apprentice	−2.91(−8.85, 3.03)	−1.61(−8.51, 5.30)	−5.36(−13.25, 2.53)	1.51(−4.82, 7.84)	1.38	0.2412
Direct cost	−0.003(−0.01, 0.005)	−0.005(−0.014, 0.004)	−0.0008(−0.01, 0.01)	−0.003(−0.01, 0.01)	0.35	0.8468
Indirect cost	0.0001(−0.005, 0.005)	−0.007(−0.01, −0.001)	−0.001(−0.01, 0.01)	−0.004(−0.01, 0.001)	1.46	0.2151
	***	***				
Zarit	−0.25(−0.38, −0.13)	−0.27(−0.42, −0.12)	−0.06(−0.23, 0.11)	−0.10(−0.23, 0.04)	5.30	0.0004***
Duration of care given	−0.003(−0.013, 0.007)	−0.004(−0.02, 0.01)	0.01(−0.01, 0.02)	0.002(−0.01, 0.01)	0.85	0.4931
Daily care given time in hrs	0.14(−0.16, 0.44)	0.17(−0.18, 0.52)	0.002(−0.40, 0.40)	0.27(−0.05, 0.59)	0.63	0.6409
Other family	*	**				
No	*Ref*	*Ref*	*Ref*	*Ref*		
Yes	2.38(0.47, 4.29)	2.90(0.68, 5.12)	−2.33(−4.87, 0.20)	0.56(−1.48, 2.59)	6.24	0.0001***
Anxiety		**				
Normal	*Ref*					
Moderate	0.13(−2.22, 2.47)	1.29(−1.43, 4.01)	0.07(−3.04, 3.18)	0.74(−1.75, 3.24)		
Severe	−2.80(−6.48, 0.87)	5.12(0.84, 9.39)	−0.18(−5.06, 4.70)	3.60(−0.31, 7.52)	4.06	0.0030**
Depression	***	***	***	***		
Normal	*Ref*	*Ref*	*Ref*	*Ref*		
Moderate	−2.73(−5.00, −0.47)	−6.02(−8.66, −3.39)	−3.30(−6.31, −0.29)	−5.11(−7.52, −2.70)		
Severe	−8.39(−13.15, −3.64)	−17.39(−22.92, −11.87)	−12.29(−18.60, −5.97)	−16.40(−21.47, −11.34)	19.20	0.0001***
Stress			**			
Normal	*Ref*	*Ref*	*Ref*	*Ref*		
Moderate	2.46(−0.45, 5.36)	3.04(−0.34, 6.41)	0.30(−3.56, 4.15)	2.34(−0.76, 5.44)		
Severe	−2.44(−6.48, 1.60)	−1.26(−5.96, 3.44)	−10.43 (−15.80, −5.06)	−1.33(−5.64, 2.988)	3.73	0.0054**
*R* ^2^	68.5	69.8	64.3	67.2		

*β* is the estimated effect of covariate, *R* ^2^ is the adjusted coefficient off determination, *CI* is confidence interval; *ref.* is the reference category, *Phy* (Physical domain), *Psy* (Psychological domain), *Soc* (Social domain), *Env* (Environmental domain)

* p < 0.05, ** p < 0.01, *** p < 0.001

The results from the multiple linear regression analysis in Table 9 indicated that a unit increase in Zarit burden score reduced caregivers’ QoL by 0.17 (95% CI: -0.28, −0.06; *p* = 0.0030), controlling for other covariates in the model. Overall QoL was 17% lower for caregivers with depression compared to those with no depression.

**Table 9:** Multiple regression results for covariates of overall quality of life

Covariates	Overall quality of life score	
	*β*	95% CI
Age in years	−0.08	(−0.15, −0.1)
Sex***		
Female	*Ref*	
Male	3.72	(1.99, 5.45)
Marital status*		
Single	*Ref*	
Married	−2.29	(−4.11, −0.47)
Proximity***		
Close	*Ref*	
Far	−4.03	(−6.01, −2.05)
Education***		
None	*Ref*	
Primary/junior high school	4.46	(1.59, 7.32)
Secondary	4.98	(2.06, 7.89)
Tertiary	10.61	(7.27,13.96)
Employment status		
None	*Ref*	
Private	0.59	(−2.63, 3.81)
Public	−1.74	(−5.30, 1.83)
Self-employed	1.59	(−0.87, 4.05)
Student/apprentice	−2.09	(−7.41, 3.22)
Direct cost	−0.003	(−0.01,0.004)
Indirect cost	−0.003	(−0.01, 0.001)
Zarit	−0.17	(−0.28, −0.06)
Duration of care given	0.0002	(−0.01, 0.01)
Daily Care given time in hours	0.15	(−0.12, 0.41)
Other family		
No	*Ref*	
Yes	0.88	(−0.83, 2.58)
Anxiety		
Normal	*Ref*	
Moderate	0.56	(−1.54, 2.65)
Severe	1.43	(−1.85, 4.72)
Depression***		
Normal	*Ref*	
Moderate	−4.29	(−6.32, −2.26)
Severe	−13.62	(−17.87, −9.37)
Stress**		
Normal	*Ref*	
Moderate	2.03	(−0.57, 4.63)
Severe	−3.87	(−7.48, −0.25)
*R* ^2^	70.1	

*β* is the estimated effect of covariate, *R* ^2^ is the adjusted coefficient off determination, *CI* is confidence interval, *ref.* is the reference category

*p < 0.05, **p < 0.01, ***p < 0.001

## Discussion

The aim of this study was to estimate the economic costs and QoL of family caregivers of schizophrenic patients attending three psychiatric hospitals in Ghana and to assess the factors associated with these two outcomes. The average costs incurred by caregivers amounted to approximately US$273 per month, with about 82% of these costs being indirect costs (in terms of lost productivity). The mean caregiver burden (measured by the ZBI) was 16.95 on a scale of 0–48, with 49% of caregivers reporting high burden. Overall, caregivers reported low QoL (28.2 out of 100).

The estimated mean monthly direct cost per month (US$30) represented about 18% of the total cost involved in caregiving. This proportion is lower than that obtained by Addo et al. [25] (21%) in their study on the cost of caregiving for mental illness (not schizophrenia specifically as in the current study) in Ghana. Our finding is also lower than that found by Zhai et al. [3] (33%) in their study on the cost of caregiving for schizophrenia in China. Although Zhai et al. [3]’s study was specific to schizophrenia, differences in the study contexts may have accounted for the differences in the proportion accounted for by direct costs, implying that access to treatments and study contexts could influence the proportion of overall costs of caring for vulnerable populations. The relative contribution of direct costs to the total economic costs further implies that caregivers bear significant indirect costs which must be taken into account in efforts to ameliorate the economic burden of mental health on families.

Similar to Knock et al. [26], the current study’s results demonstrate that caregivers of people with schizophrenia bear levels of other types of psychological and social burdens that are difficult to quantify. The current study found that caregivers of schizophrenics reported lower QoL compared to previous studies [16, 27] conducted in Chile and France. Factors such as caregiver burden and depression were associated with lower QoL, whereas support from other family members and education were associated with higher QoL. Sex was also significantly associated with QoL, with males reporting higher QoL than females. Further, 61% of caregivers who reported higher burden were females and 39% were males. Ohaeri [28] argues that caregiving roles are primarily undertaken by female family members who bear much of the burden (e.g., psychological effect) of care in Nigeria. Similarly, Papastavrou et al. [29] found that females were more burdened than males and experienced chronic stress because of caregiving responsibilities for schizophrenics in Cyprus. Sex differences in the burden of care have also been reported in studies examining elderly care [30]. Such findings reflect broader societal realities and require more in-depth investigation.

There are limitations to the study that are worth noting. First, it was not possible to determine whether the caregiver received any assistance, in cash or in kind from other members of the family or the patient; thus, the cost burden may not be entirely attributed to the main caregiver. Second, it was not possible to conduct more nuanced analysis to assess possible underlying reasons (e.g., coping styles) for some of the differences observed. Nonetheless, the current study adds to the sparse literature on the economic and social costs of mental disorders in sub-Saharan Africa.

## Conclusions

The study findings highlight the significant burden of caring for people with schizophrenia on family caregivers in Ghana. As the country considers deinstitutionalizing mental health care, it is important that measures to alleviate the direct costs on caregivers are taken into account. Currently, the National Health Insurance Scheme (NHIS) does not cover services for schizophrenia or other mental disorder, deepening the burden of families. One possible avenue to alleviate the costs incurred by caregivers of people with mental illness would be to extend social protection programs, such as the Livelihood Empowerment Against Poverty (LEAP) program (which provides cash transfers to vulnerable groups), to caregivers to cushion the effects of the shocks of the direct costs. Study findings also underscore the significant non-quantifiable burdens, such as emotional stress, that are borne by caregivers and that affect their quality of life. The quality of life of caregivers of patients with mental disorders should be considered in health policies related to mental illness.

## Abbreviations

DALYs: Disability-adjusted life-years
DASS: Depression anxiety stress scale
GHS: Ghanaian Cedi
ICD-10: International Classification of Disease, 10^th^ edition
LEAP: Livelihood empowerment against poverty
LMICs: Low and middle income countries
NHIS: National Health Insurance Scheme
PPS: Probability proportionate to size
QoL: Quality of Life
UK: United Kingdom
US$: United States Dollar
WHO: World Health Organisation
WHOQOL: WHO Quality of Life
WHOQOL-BREF: abridged WHO Quality of Life
YLD: Years lived with disability
ZBI: Zarit Burden Interview
